# Isolated Bulbar Palsy: A Rare Presentation of Neurosarcoidosis

**DOI:** 10.7759/cureus.7010

**Published:** 2020-02-16

**Authors:** Hammad S Chaudhry, Fateen Ata, Mohamed Abdelghani, Rashid Kazman, Ali Rahil

**Affiliations:** 1 Internal Medicine, Hamad Medical Corporation, Doha, QAT; 2 Cardiology, Heart Hospital, Hamad Medical Corporation, Doha, QAT; 3 Internal Medicine, Hamad General Hospital, Doha, QAT

**Keywords:** sarcoidosis, neurosarcoidosis, ivig, steroids, bulbar palsy

## Abstract

Sarcoidosis, which is a non-caseating granulomatous chronic inflammatory disease, can affect virtually any organ system, including the central nervous system (CNS). Very rarely, patients may present solely with neurosarcoidosis. It commonly presents with unilateral or bilateral seventh nerve palsy. Rarely it can present as dangerous progressive bulbar palsy and is a diagnostic and clinical challenge. We present a case of sarcoidosis with a unique presentation of isolated bulbar palsy. A 38-year-old male presented to the emergency with a sudden onset of dysphonia followed by dysphagia for both solids and liquids for one week and 5 kg weight loss in two months. The rest of the CNS exam was unremarkable. On labs, he had hypercalcemia and suppressed parathyroid hormone (PTH) intact. Detailed radiological investigations, lab tests, and lymph node biopsy helped confirm the diagnosis of neurosarcoidosis. The patient did not respond to first-line steroid therapy and hence received intravenous immunoglobulin (IVIG) subsequently with adequate response and complete neurologic recovery, confirmed by a follow-up visit.

## Introduction

Sarcoidosis is a multi-system chronic inflammatory disease with non-caseating granuloma formation, which can affect virtually every organ system. The involvement of the central nervous system (CNS) is a relatively rare phenomenon. Approximately 5%-10% of patients with sarcoidosis develop neurosarcoidosis, and only 1% of patients present solely with neurosarcoidosis [1]. It commonly presents with unilateral or bilateral seventh nerve palsy; leptomeningitis is also a common presentation [1-2]. In rare instances, neurosarcoidosis can present as dangerously progressive bulbar palsy, which is a diagnostic and clinical challenge. In patients with neurosarcoidosis, less than 4% of patients have involvement of the cranial nerve nine, ten, and twelve, leading to dysfunction of the throat muscles, tongue muscles, palatine muscles and those of the vocal cords [3]. Here we present a case of sarcoidosis with predominantly bulbar involvement.

## Case presentation

A 38-years-old male from the Indian sub-continent presented to the emergency department with sudden onset of dysphonia followed by dysphagia for both solids and liquids for one week. The patient also reported 5 kg of unintentional weight loss over two months. He did not give a history of eating any spoiled food in the recent past. His voice had become nasal in quality, and drinking more water frequently led to regurgitation of the liquid through the nose, resulting in cough. His symptoms started abruptly without any associated limb weakness. On examination, he was vitally stable, the power in all four limbs was normal, and the reflexes were intact. His planters were bilaterally down-going, and there were no cerebellar signs. On cranial nerve examination, the patient had a deviation of the uvula towards the left side, and there was some palatal droop. The rest of the exam was unremarkable except for the presence of palpable lymph nodes in the right inguinal region.

Basic metabolic panel at the initial encounter revealed mild hypercalcemia (corrected calcium 2.62 mmol); however, the rest of the electrolytes were within the normal limits. The intact parathyroid hormone (PTH) level was 6.6 picograms per milliliter (reference range: 15 - 65) with low vitamin D (Table 1).

**Table 1:** Comparison of labs done initially, after steroids, after IVIG, and at follow-up PTH: Parathyroid hormone; IVIG: Intravenous immunoglobulins.

Investigation	Initial	5 days post Steroids	5 days post IVIG	At Follow-up
Serum Calcium (mmol/L)	2.62	2.51	2.53	2.50
PTH (pg/ml)	6.6	-	-	-
Vitamin D (ng/ml) (<20 Deficiency, 21-29 Insufficiency, >30 Optimum)	16	-	-	14

**Figure 1:**
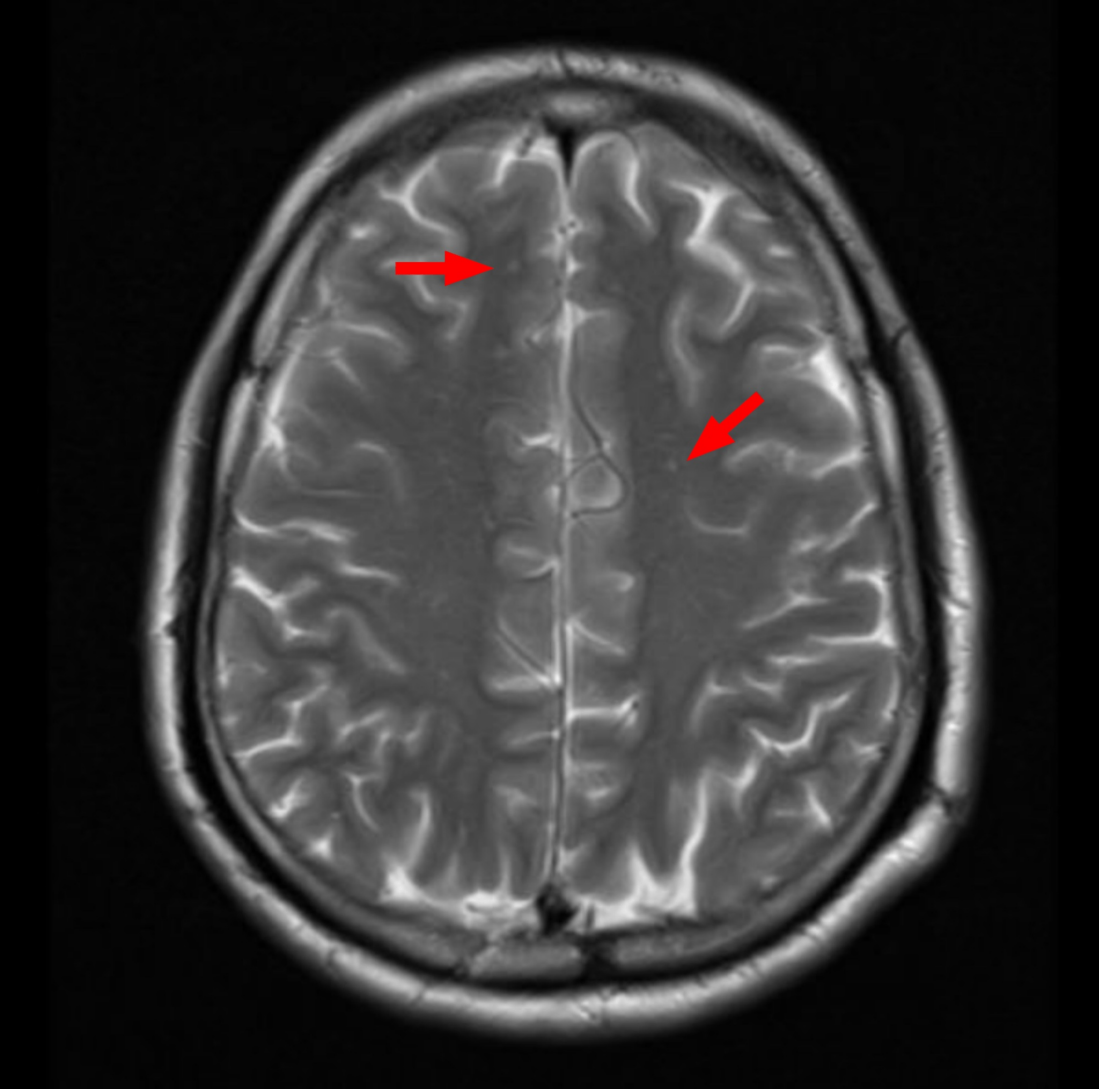
Magnetic resonance imaging (MRI) of the brain Red arrows: multiple small, high signal foci in white matter.

Chest and neck X-ray did not elucidate any anatomical pathology as a possible explanation. The initial neurological impression was of bulbar palsy. Due to the presence of neurologic symptoms, stroke was thought of, which was ruled out by a computed tomography (CT) head, followed by a magnetic resonance imaging (MRI) of the head, which showed some hyperintense non-specific lesions in the frontal and the parietal lobe (Figure 1). They were considered nonspecific because the patient was hypertensive for the past seven years. Neurologic tuberculosis was another possibility, as the patient originally belonged, and had frequent travels, to a region with high endemicity. Tuberculosis was excluded by a negative QuantiFERON TB test (Cellestis Limited, Carnegie, Victoria, Australia). A negative antinuclear antibody (ANA) and antineutrophil cytoplasmic antibody (ANCA) test which were done after the initial blood investigations were inconclusive; it came back negative, making a diagnosis of connective tissue disease and vasculitis less likely.

**Figure 2:**
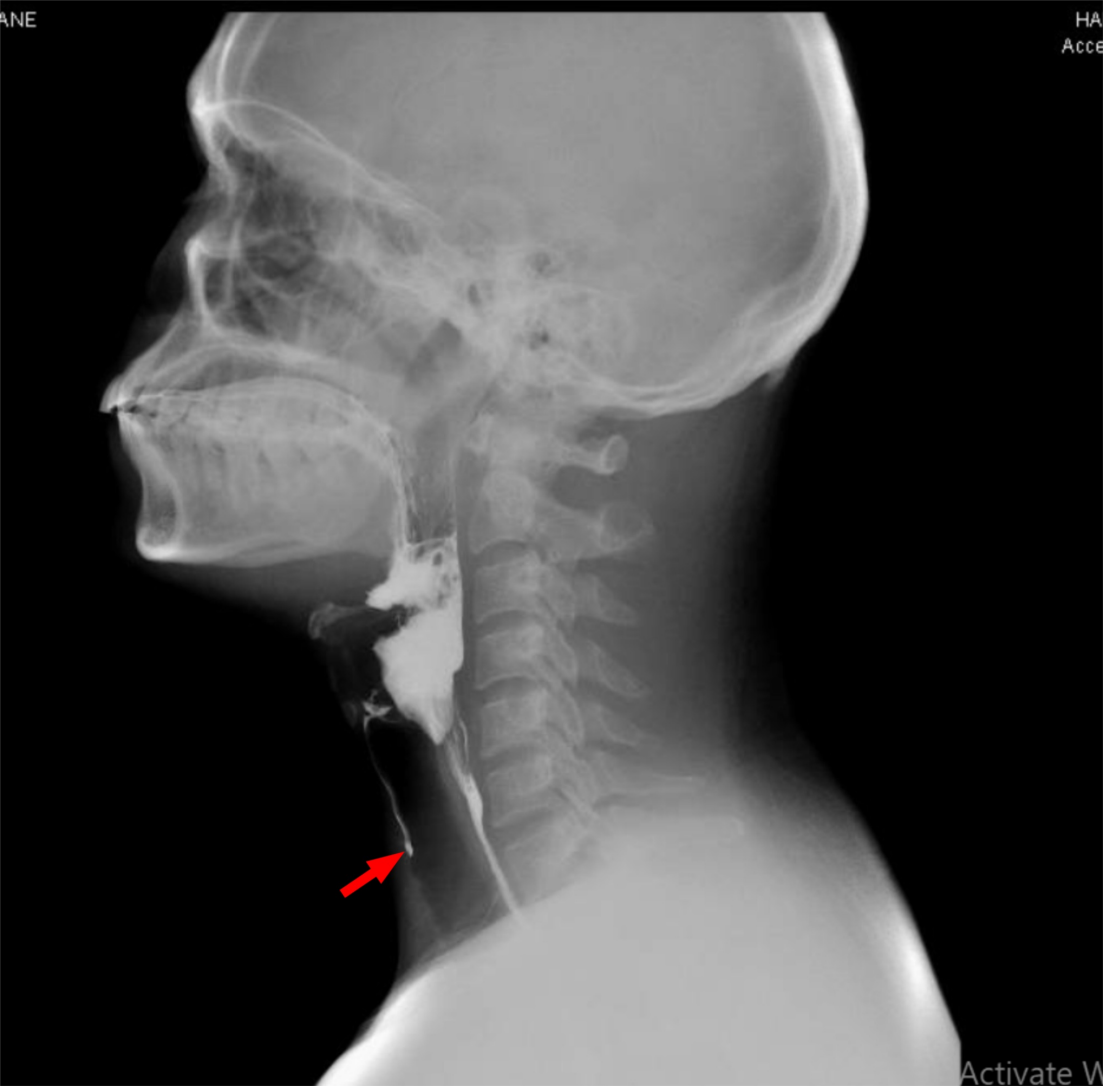
Initial video-fluoroscopic swallowing study Red arrow: contrast entering airway indicating ineffective swallowing.

**Figure 3:**
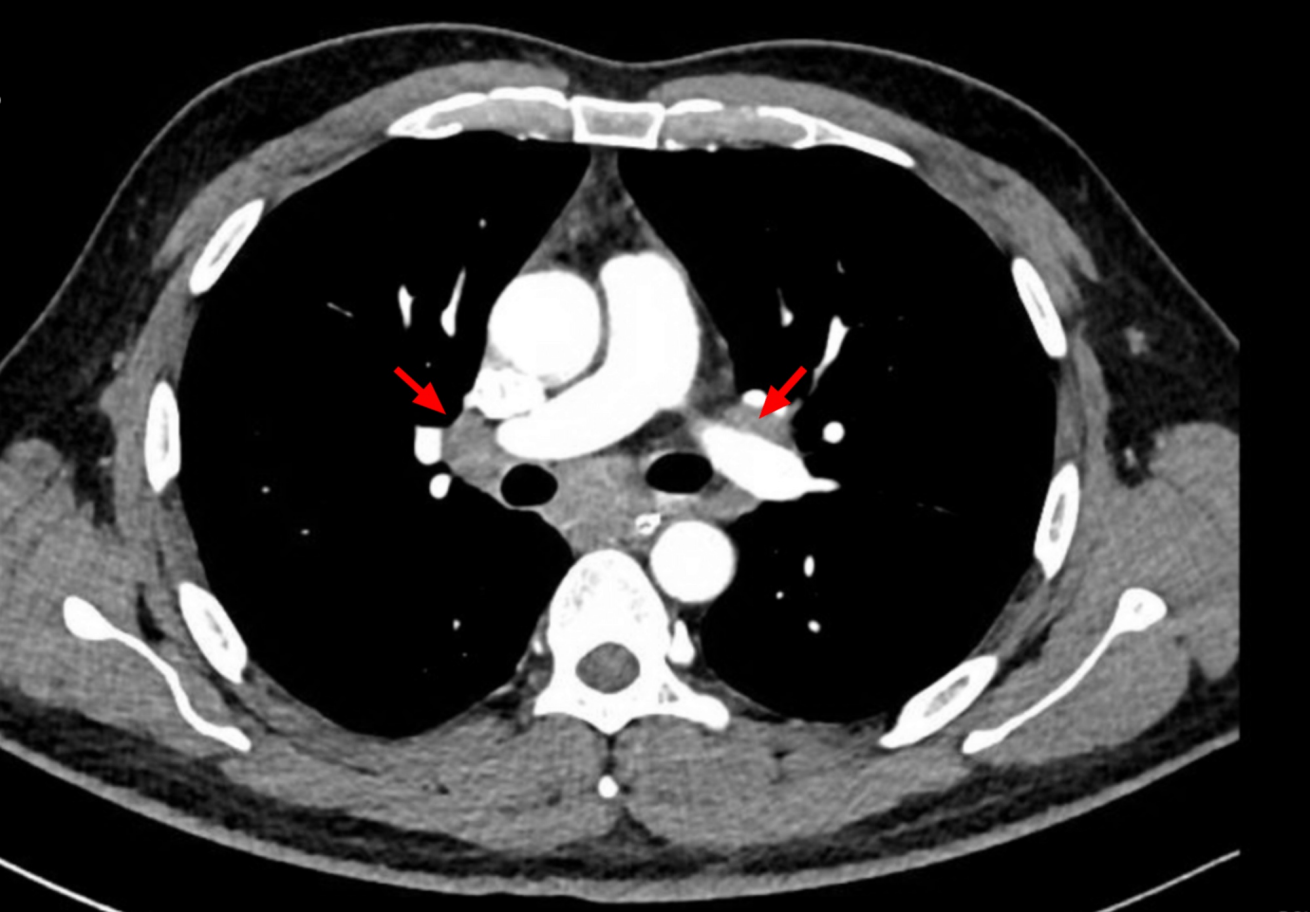
Computed tomography (CT) scan of the thorax Red arrows: paratracheal and hilar lymph nodes.

Video-assisted modified barium swallow (done to assess the swallowing capacity of the patient) showed that the patient had mild palatal abnormality with the partial inability of the epiglottis to cover the respiratory tract, leading to micro aspirations which prompted the insertion of the nasogastric tube to prevent aspirations (Figure 2). The patient’s voice became hoarser and nasal over the course of his hospital stay, and his calcium levels were rising gradually. A subsequent contrast-enhanced thoracic CT scan which was ordered while keeping lymphomas as a possible cause of his symptomatology, showed multiple lymph nodes in the paratracheal and hilar regions (Figure 3).

**Figure 4:**
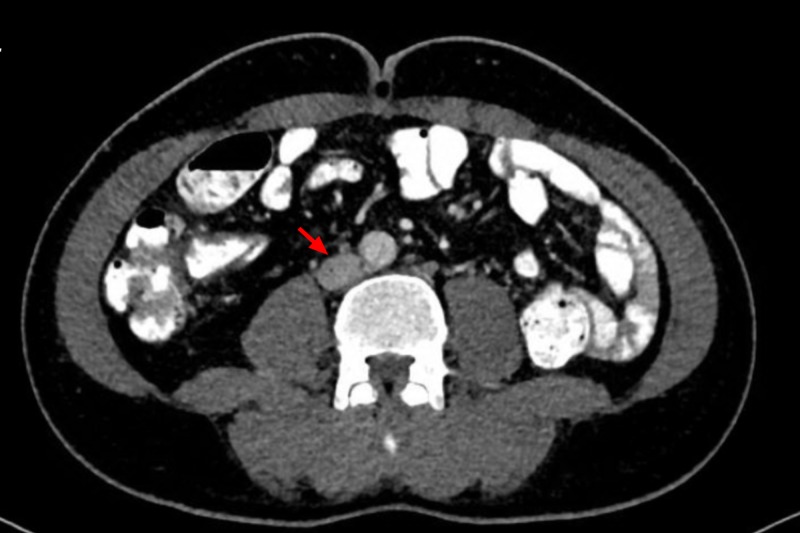
Computed tomography (CT) scan of the abdomen Red arrows: para-aortic lymph node.

**Figure 5:**
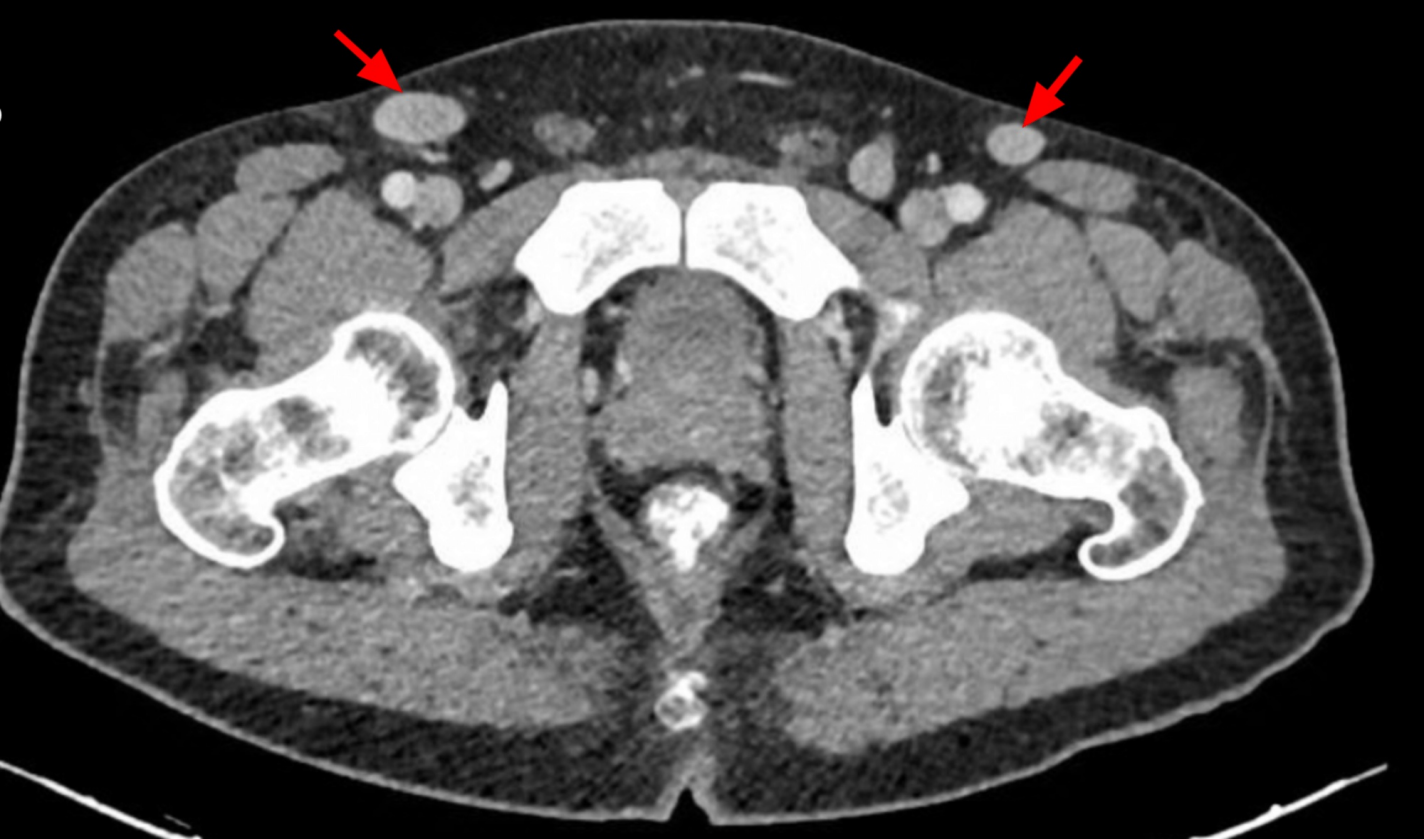
Computed tomography (CT) scan of the pelvis Red arrows: inguinal lymph nodes.

**Figure 6:**
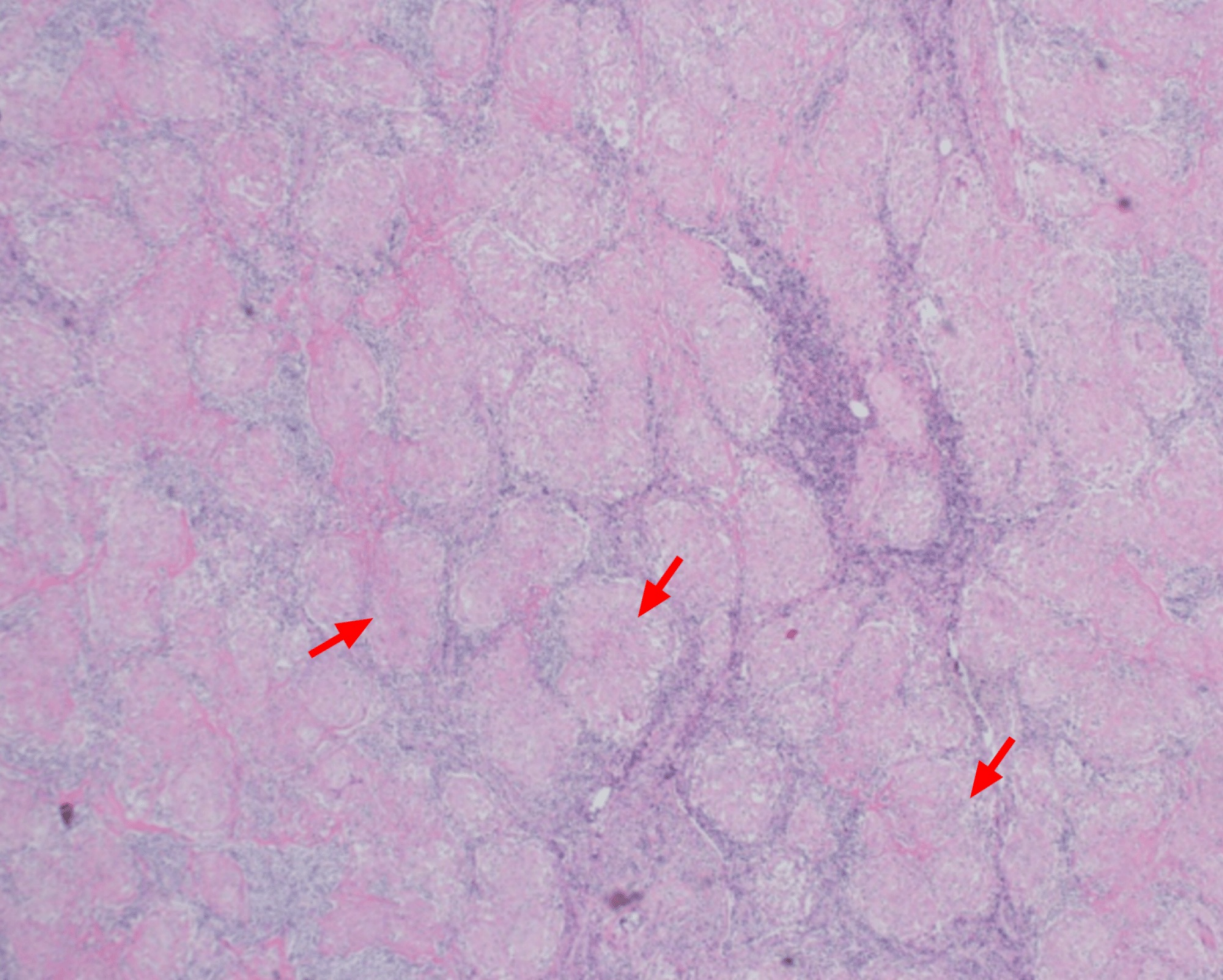
Multiple non-caseating granulomas from the inguinal lymph node (red arrows)

**Figure 7:**
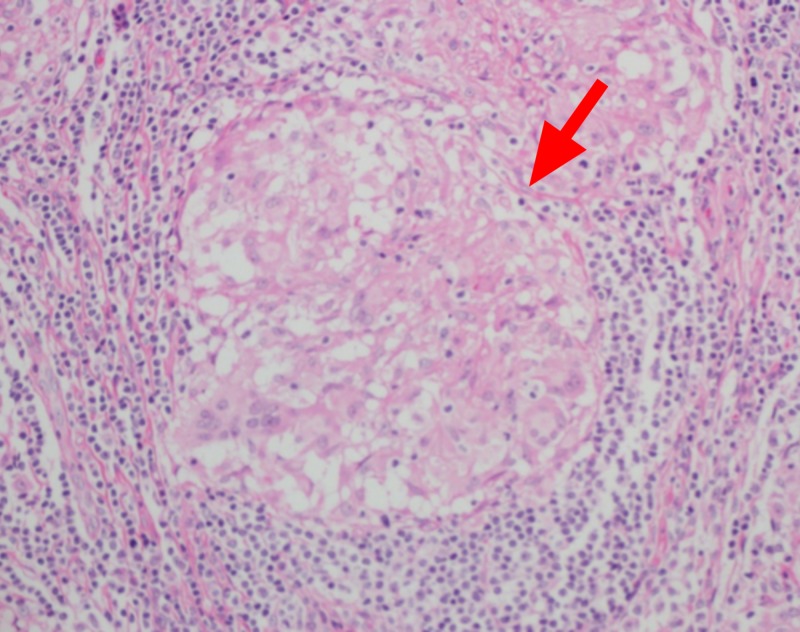
Non-caseating granuloma (red arrow)

A contrast-enhanced CT scan of the abdomen and pelvis elucidated multiple para-aortic, iliac, and superficial and deep inguinal lymph nodes (Figures 4-5). Inguinal lymph nodes, being sizeable for an excisional biopsy, were sent for histopathological examination, revealing a granulomatous inflammation without caseation with a negative acid-fast bacillus smear and polymerase chain reaction (PCR) (Figures 6-7). Given the exclusion of other possible conditions and suggestive biopsy findings, the diagnosis of sarcoidosis was established at this point. Although asymptomatic, the patient’s calcium level was still elevated, which was managed by intravenous normal saline.

Pulse steroid therapy was started with intravenous methylprednisolone for five days. On the third day of pulse steroids, the patient’s calcium levels returned to baseline, as seen in Table 1; however, that patient’s voice and swallowing did not improve even after the completion of the steroid therapy. At this point, a trial of intravenous immunoglobulin (IVIG) at 0.4 g/kg/body weight was started. After two doses of the IVIG, the patient showed significant improvement in his swallowing capacity and dysphonia, and his calcium levels remained normal.

**Figure 8:**
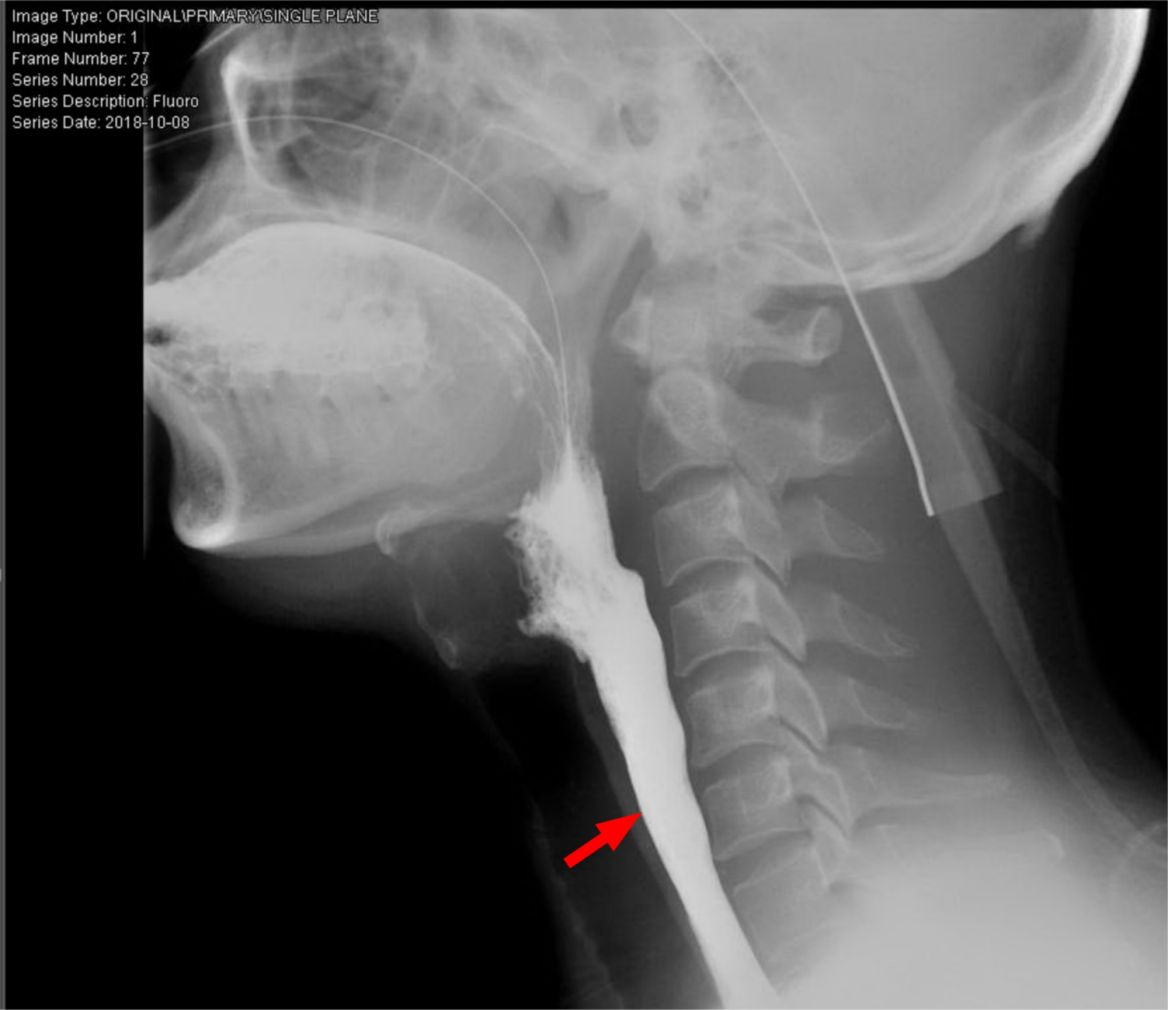
Repeat video-fluoroscopic swallowing study Red arrow: contrast going into the esophagus indicating effective swallowing.

A repeat video fluoroscopy showed marked improvement in his swallowing capabilities, which led to the removal of the nasogastric tube. The patient completed the course of IVIG for five days and was discharged from the hospital asymptomatic with a follow-up appointment (Figure 8).

Follow-up visits

One month after discharge from the hospital, the patient followed up with the pulmonology team. The patient was completely asymptomatic. He was continued on regular steroids with tapering over the next few months. On a subsequent visit with pulmonology after another month, the patient was asymptomatic and did not complain of change in voice or dysphagia. He had a follow-up visit with the neurology four months after his initial discharge, where a complete neurological assessment was done and found within the normal range. The patient had stopped taking steroids at this point according to the schedule; he was kept on regular follow up.

## Discussion

Neurosarcoidosis, presenting with isolated bulbar palsy, is a rare occurrence. The mean age of presentation of neurosarcoidosis is about 33-41 years of age [4]. Less than 1% of cases of sarcoidosis present with isolated CNS involvement [5]. Sarcoidosis may present with a wide array of presentations such peripheral neuropathy, myopathy, mono neuritis multiplex, Guillain barre like syndrome, bilateral facial nerve palsy, and last but not least as leptomeningitis while the parenchymal spread of the sarcoidosis involves the base of the brain making cranial nerve susceptible [4,6]. Neurosarcoidosis may present in as much as 5%-10% cases of sarcoidosis and of the cranial nerves facial, and the ophthalmic nerves are the most likely to be involved [2]. There are myriads of presentations of the neurological involvement but presenting as progressive bulbar palsy is extremely rare [7]. The diagnosis of neurosarcoidosis is challenging, and the management of the disease is as difficult.

Diagnosis of this condition is challenging and needs multiple investigations to say with certainty that it is, in fact, neurosarcoidosis and not some other entity. Radiological investigations looking for supportive features such as enlarged lymph nodes or organomegaly with the use of CT scan, MRI, and 18fluoro-2-deoxyglucose-positron emission tomography (FDG-PET) scans are frequently required. In addition, biochemical markers such as angiotensin-converting enzyme levels, and inflammatory markers may help in the diagnosis, yet none of them can individually diagnose the disease [6]. A PET scan utilizing FDG may show characteristic “Panda sign” on chest imaging, however, it may also be seen in other conditions such as malignancy, and tuberculosis, failing to provide a definitive diagnosis [3]. However, newer PET scan modalities that utilize Flour thymidine may help to differentiate sarcoidosis and malignancies and are known to point the site for biopsy with much better accuracy compared to the MRI and CT scans [3]. There is a poor correlation between the imaging findings and clinical signs and symptoms if the central nervous system is involved, and so for diagnosis, one can not only rely on imaging alone [5]. A range of diagnostic tools is required to diagnose neurosarcoidosis, including radiological studies combined with biopsy of the accessible areas when possible. Angiotensin-converting enzyme (ACE) levels are positive in sarcoidosis, but it is not specific; moreover, ACE levels from the bronchoalveolar lavage (BAL) are more specific for the sarcoidosis compared to ACE levels in the plasma [2]. In our case, we tried to get the biopsy sample from the mediastinal lymph node but we failed to obtain enough tissue, however, we obtained biopsy from the inguinal lymph node which showed characteristic granuloma’s making diagnosis obvious, we could not obtain ACE levels as the test was not available in the country. Biopsy with suggestive imaging and clinical picture after ruling out other plausible diagnoses helped us in making the diagnosis of neurosarcoidosis.

There are no randomized controlled trials that evaluated any specific treatment for neurosarcoidosis, however, based on case reports and expert opinion corticosteroids have been used as the first line of therapy [6]. Other agents that can be used in case of inadequate response to steroids include immunomodulators and cytotoxic drugs such as methotrexate, azathioprine, rituximab, adalimumab, infliximab, cyclophosphamide, and cyclosporine [4,6]. Steroid- resistant neurosarcoidosis has shown response to anti-tumour necrosis factor (TNF) drugs like infliximab [8]. However, in one research, steroid-resistant neurosarcoidosis which failed to respond to infliximab alone responded to the addition of IVIG to the regimen. In different presentations of the neurosarcoidosis, such as multiple conduction blocks and small fiber neuropathy, which were resistant to first-line and second-line therapeutic agents responded to IVIGs [9]. Our patient responded very well to the addition of IVIG supporting the result of other reported cases [7,10].

The mortality associated with this malady is 5% [6]. However; understanding the effect of IVIG on mortality needs more studies. Our case highlights the importance of considering sarcoidosis as a possible cause of bulbar palsy; timely efforts are needed to confirm the diagnosis and treat the condition to prevent any permanent neurological damage.

## Conclusions

Neurosarcoidosis is a diagnostic possibility that can explain a challenging case of undiagnosed but potentially treatable bulbar palsy; therefore, it should be considered and actively looked for with the use of appropriate imaging modalities and tissue diagnosis where possible. IVIG therapy has demonstrated adequate response in steroid-resistant cases as per previously reported cases and our case report consolidates this approach; however, a greater level of evidence is needed to support it.
